# Prediction of human *O-*linked glycosylation sites using stacked generalization and embeddings from pre-trained protein language model

**DOI:** 10.1093/bioinformatics/btae643

**Published:** 2024-10-24

**Authors:** Subash Chandra Pakhrin, Neha Chauhan, Salman Khan, Jamie Upadhyaya, Moriah Rene Beck, Eduardo Blanco

**Affiliations:** Department of Computer Science and Engineering Technology, University of Houston-Downtown, Houston, TX 77002, United States; School of Computing, Wichita State University, Wichita, KS 67260, United States; Department of Computer Science, The University of Texas at Austin, Austin, TX 78712, United States; Department of Computer Science and Engineering Technology, University of Houston-Downtown, Houston, TX 77002, United States; Department of Chemistry and Biochemistry, Wichita State University, Wichita, KS 67260, United States; Department of Computer Science, University of Arizona, Tucson, AZ 85721, United States

## Abstract

**Motivation:**

*O-*linked glycosylation, an essential post-translational modification process in *Homo sapiens*, involves attaching sugar moieties to the oxygen atoms of serine and/or threonine residues. It influences various biological and cellular functions. While threonine or serine residues within protein sequences are potential sites for *O-*linked glycosylation, not all serine and/or threonine residues undergo this modification, underscoring the importance of characterizing its occurrence. This study presents a novel approach for predicting intracellular and extracellular *O-*linked glycosylation events on proteins, which are crucial for comprehending cellular processes. Two base multi-layer perceptron models were trained by leveraging a stacked generalization framework. These base models respectively use ProtT5 and Ankh *O-*linked glycosylation site-specific embeddings whose combined predictions are used to train the meta-multi-layer perceptron model. Trained on extensive *O-*linked glycosylation datasets, the stacked-generalization model demonstrated high predictive performance on independent test datasets. Furthermore, the study emphasizes the distinction between nucleocytoplasmic and extracellular *O-*linked glycosylation, offering insights into their functional implications that were overlooked in previous studies. By integrating the protein language model’s embedding with stacked generalization techniques, this approach enhances predictive accuracy of *O-*linked glycosylation events and illuminates the intricate roles of *O-*linked glycosylation in proteomics, potentially accelerating the discovery of novel glycosylation sites.

**Results:**

Stack-OglyPred-PLM produces Sensitivity, Specificity, Matthews Correlation Coefficient, and Accuracy of 90.50%, 89.60%, 0.464, and 89.70%, respectively on a benchmark NetOGlyc-4.0 independent test dataset. These results demonstrate that Stack-OglyPred-PLM is a robust computational tool to predict *O-*linked glycosylation sites in proteins.

**Availability and implementation:**

The developed tool, programs, training, and test dataset are available at https://github.com/PakhrinLab/Stack-OglyPred-PLM.

## 1 Introduction

*O-*linked glycosylation is an essential post-translational modification (PTM) in human biology (Ohtsubo and Marth 2006). This PTM occurs through the addition of glycans to threonine (T) or serine (S) amino acids of protein sequences (Yang 2011). The presence of *O-*linked glycosylation sites profoundly influences protein structure and function, contributing to a spectrum of physiological and pathological outcomes, including disruptions in intercellular communication, cellular dysfunction, immune deficiencies, hereditary disorders, congenital disorders of glycosylation, and various cancers (Boskovski *et al.* 2013, Wolfert and Boons 2013, Campos *et al.* 2015, Chen *et al.* 2015). Hence, the accurate identification of *O-*linked glycosylation sites has significant implications for therapeutic interventions in human health.

There are two major kinds of *O-*linked glycosylation in humans, namely (i) nucleocytoplasmic *O-*linked glycosylation (GlcNAc) and (ii) extracellular *O-*linked glycosylation (GalNAc). The distinction between nucleocytoplasmic and extracellular *O-*linked glycosylation is critical in understanding the complexity of glycosylation processes within proteins. Nucleocytoplasmic *O-*linked glycosylation involves the addition of *O-*linked *N*-acetylglucosamine (*O*-GlcNAc) moieties to the serine and threonine residues of nuclear and cytoplasmic proteins (Hart *et al.* 2011). This process is catalyzed by a single enzyme known as *O*-GlcNAc transferase (OGT). OGT plays crucial roles in various cellular processes, such as transcription, signaling, and protein stability regulation. On the other hand, extracellular *O-*linked glycosylation, often called GalNAc-type glycosylation, occurs outside the cell (extracellular space) and involves adding N-acetyl galactosamine (GalNAc) residues to secreted or cell surface proteins (Tran and Ten Hagen 2013, Bagdonaite *et al.* 2020, Nielsen *et al.* 2022). Unlike nucleocytoplasmic *O-*glycosylation, extracellular *O-*glycosylation is mediated by multiple enzymes across multiple pathways.

Mass spectrometry is the primary experimental method for identifying *O-*linked glycosylation sites. While highly reliable, this experimental approach is often encumbered by sensitivity, labor intensiveness, and costliness. Consequently, the adoption of machine learning (ML) and deep learning (DL) methodologies emerge as prominent solutions for characterizing *O-*linked glycosylation sites efficiently and effectively.

Several different *O-*linked glycosylation site prediction models currently exist. The most utilized program remains NetOGlyc-4.0 (Steentoft *et al.* 2013). However, like most prediction models the input features are hand-crafted which can result in bias and are highly dependent on the quality of input data (Julenius *et al.* 2005, Li *et al.* 2006, Caragea *et al.* 2007, Chen *et al.* 2008, Li *et al.* 2015, Taherzadeh *et al.* 2019, Huang *et al.* 2021, Mohl *et al.* 2021, Pakhrin *et al.* 2021, Pakhrin 2022, Zhu *et al.* 2022, Hu *et al.* 2024). Moreover, the disparity between nucleocytoplasmic and extracellular *O-*linked glycosylation processes has been overlooked in this program. In addition, recent advancements in large protein language models (PLMs) and the context-based representation derived from these models, particularly concerning nucleocytoplasmic and extracellular localization, have not been explored for predicting *O-*linked glycosylation sites. It is noteworthy that PLM embeddings are used in pioneering research across various areas, such as predicting *N-*linked glycosylation PTM (Pakhrin *et al.* 2023a,b), phosphorylation PTM (Pakhrin *et al.* 2023a,b), SUMOylation PTM (Palacios *et al.* 2024), and protein–ligand binding (Littmann *et al.* 2021).

To comprehensively address these limitations, we present a novel *O-*linked glycosylation predictor. Unlike previous approaches, our predictor utilizes stacked generalization (SG) (Wolpert 1992), a DL technique leveraging PLMs’ *O-*linked glycosylation site-specific embedding to improve accuracy and robustness. Hence, we refer to our model as Stack-OglyPred-PLM (**S**tacked generalization-based ***O****-*linked **g**lycosylation sites **p**rediction using a **p**rotein **l**anguage **m**odel). More importantly, we have considered the distinguishing mechanisms of *O-*linked glycosylation that occur in different cellular compartments, namely the nucleus and cytoplasm, versus the extracellular space. Therefore, we chose 1821 nucleocytoplasmic *O-*linked glycoproteins from the dbPTM dataset, and likewise, we selected 154 extracellular *O-*linked glycoproteins from the NetOGlyc-4.0 dataset. Our approach not only improves prediction accuracy but also enhances our understanding of the functional implications of *O-*linked glycosylation in various biological processes. Through this work, we advance the field of glycoproteomic and pave the way for deeper insights into the role of *O-*linked glycosylation in health and disease. Furthermore, such protein segregation is essential because PLMs can differentiate between residues in the nucleocytoplasmic region and those in extracellular space, a capability facilitated by inherent biases in amino acid composition across these compartments.

We developed two separate predictive *O-*linked glycosylation models: (i) the nucleocytoplasmic and (ii) the extracellular. The nucleocytoplasmic model underwent training and testing using nuclear and cytoplasmic *O-*linked glycoproteins, while the extracellular model underwent training and testing with *O-*linked glycoproteins located in the extracellular space (cell surface or secreted). Each model uses a stacked generalization method, with the base model utilizing *O-*linked site-specific embeddings from ProtT5 (Elnaggar *et al.* 2022) and Ankh (Elnaggar *et al.* 2023) protein language models processed by respective multi-layer perceptron (MLP) architectures. The predictions from these base models were then combined to train the MLP meta-model. While comparing our extracellular model’s independent test dataset result with the NetOGlyc-4.0 method, our extracellular model yielded MCC, ACC, SN, and SP values of 0.464, 89.7%, 90.5%, and 89.6%, respectively, which is much better than the NetOGlyc-4.0 predictor (Steentoft *et al.* 2013). Hence, our models can be used for the detection of *O-*linked glycosylation in proteins, expediting the discovery of novel *O-*linked glycosylation sites in proteins.

## 2 Materials and methods

### 2.1 Datasets

We used three datasets, namely dbPTM (Li *et al.* 2022) for nucleocytoplasmic *O-*linked glycosylation, extracellular NetOGlyc-4.0 (Steentoft *et al.* 2013), and extracellular GalNAc-T (Nielsen *et al.* 2022) for analysis.

#### 2.1.1 dbPTM nucleocytoplasmic dataset

The dbPTM repository comprises 3314 *O-*linked glycoproteins (Li *et al.* 2022). Given that *O*-GlcNAc transferase (OGT) is responsible for *O*-GlcNAcylation, a post-translational modification where a single N-acetylglucosamine (GlcNAc) molecule is added to serine or threonine residues of nuclear and cytoplasmic proteins, we aimed to focus solely on proteins residing in these cellular compartments to ensure biological relevance. Therefore, we subjected all 3314 *O-*linked glycoproteins to DeepLoc 2.0 (Thumuluri *et al.* 2022) subcellular localization software, which classified 2231 proteins as nucleus and cytoplasm dwellers.

Considering potential redundancy among protein sequences and to prevent overestimation of prediction accuracy while maintaining diversity, we used psi-cd-hit software with a 30% threshold (Huang *et al.* 2010). This criterion preserved only one protein when two or more proteins had 30% or more sequence similarity. After this filtration, we were left with 1821 proteins from 2231 proteins. From these 1821 proteins, we allocated 1638 proteins for training and 183 proteins for independent testing purposes. Next, we identified 5724 positive “S/T” *O-*linked glycosylated sites from 1683 proteins marked as positive in the dbPTM dataset. From this same subset of 1683 proteins, we identified 232 286 “S/T” sites as negative sites other than the dbPTM *O-*linked positive training dataset.

Given the considerable imbalance between positive and negative sites, we implemented a random under-sampling technique on the training dataset. This involved randomly selecting 5724 “S/T” negative sites from the pool of 232 285 negative sites. These balanced datasets were then utilized as the training set for our nucleocytoplasmic model. For the independent test dataset, we isolated 1062 positive “S/T” *O*-linked glycosylation sites from the 183 proteins. Correspondingly, we extracted 27 031 negative “S/T” *O*-linked glycosylation sites, excluding the positive *O*-linked glycosylation sites, from the same 183 proteins. For further details on the dataset in training and testing nucleocytoplasmic *O-*linked glycosylation sites, please refer to Table 1.

**Table 1.** Positive and negative nucleocytoplasmic *O*-linked glycosylation sites for training and independent testing.

Dataset	Number of proteins	Positive	Negative
Training	1638	5724	5724
Independent test	183	1062	27 031

#### 2.1.2 NetOGlyc-4.0 dataset

We developed a robust model specifically tailored to predict extracellular (GalNAc) or mucin-type *O-*linked glycosylation. To construct this model, we utilized 476 unambiguous glycoproteins obtained from the NetOGlyc-4.0 predictor (Steentoft *et al.* 2013). Given that GalNAc modifications predominantly occur in the extracellular region of cells, we subjected these 476 glycoproteins to further analysis using DeepLoc 2.0 (Thumuluri *et al.* 2022). Among them, DeepLoc 2.0 identified 174 proteins as extracellular, which were then selected for further investigation. Recognizing the potential presence of redundant and homologous proteins, we used psi-cd-hit software with a 30% cutoff to eliminate duplicates and retain one representative protein from each group (Huang *et al.* 2010). Consequently, we obtained 154 glycoproteins after applying the filtering process. Out of the 154 proteins available, we assigned 137 for training and 17 for independent testing. Within the training set, we identified 582 positive “S/T” *O-*linked glycosylation sites, along with 15 831 negative “S/T” sites, excluding the 582 positive instances. Given the substantial disparity between positive and negative training samples, we used an under-sampling technique, randomly selecting 582 negative sites from the pool of 15 831 negative sites. This serves as the training dataset for the extracellular model. In addition, we extracted 85 positive “S/T” sites and 1986 negative “S/T” sites (apart from the independent positive samples) from the 17 proteins designated for independent testing. Table 2 shows details about the number of training and independent testing sites. The third GalNAc-T (Nielsen *et al.* 2022) dataset used in our study is described in the Supplementary Materials. Furthermore, positive and negative sites are distinct in both training and independent testing phases, and vice versa, across all datasets used in this study.

**Table 2.** Positive and negative extracellular *O*-linked glycosylation sites for training and independent testing (derived from NetOGlyc-4.0).

Dataset	Number of proteins	Positive	Negative
Training	137	582	582
Independent test	17	85	1986

### 2.2 Feature encoding

The core process in research pipelines across computer vision, natural language processing, and bioinformatics (Pakhrin 2022) revolves around converting various data types as images, tokenized words, DNA, or RNA—into feature vectors that represent them effectively. Similarly, in proteomics research, it’s essential to establish methodologies for encoding amino acids into corresponding features to facilitate subsequent analysis using machine learning or deep learning algorithms. Recently, protein language models, trained on extensive protein corpora, have demonstrated their capability to generate biologically relevant features. Hence, for this study, we utilized two self-supervised trained models, ProtT5 and the Ankh protein language model, to produce contextualized embeddings at the per-residue level.

#### 2.2.1 Per-residue contextualized embeddings from ProtT5

The ProtT5-XL-UniRef50 (ProtT5) protein language model was utilized to generate contextualized embeddings for sites of interest (“S/T”) (Elnaggar *et al.* 2022). ProtT5 operates as a self-supervised learning model, built upon the T5 architecture (Raffel *et al.* 2020). This self-supervised approach allows the model to learn directly from the data, generating its signals for supervision without relying on external labels. In addition, ProtT5 is trained using a vast dataset comprising 2.5 billion unlabeled protein sequences sourced from the BFD (Steinegger *et al.* 2019) and UniRef50 (UniProt 2021) databases. The entire protein sequence is inputted into the pre-trained ProtT5 PLM to produce contextualized embeddings for each residue. Following this, embeddings corresponding to the sites of interest (“S/T”) are isolated and fed into the MLP architecture. Moreover, the Supplementary Section explains the procedure for extracting contextualized embeddings for each residue from the Ankh PLM (Elnaggar *et al.* 2023).

### 2.3 Architecture of Stack-OglyPred-PLM

The Stack-OglyPred-PLM uses the stacked generalization (meta-ensemble) technique, which involves training two distinct MLP models using ProtT5 and Ankh PLM embeddings. The prediction probability generated by each base model is concatenated and utilized to train a straightforward MLP meta-model. This meta-model learns to effectively integrate the prediction scores from base models, achieving an optimal balance between the strengths and weaknesses of each base model.

#### 2.3.1 ProtT5 base model

Initially, the complete amino acid sequences of the proteins were inputted into the ProtT5 encoder, yielding embeddings with a dimension of 1024 for each amino acids within the proteins. However, only the 1024 features corresponding to the site of interest (“S/T”) were utilized. To illustrate, if a protein comprises 345 amino acids and the *O-*linked glycosylated site resides at the 87th position then the ProtT5 PLM model would generate a matrix of size 345 × 1024. From this matrix, only the 87th feature vector of length 1024 would be extracted for analysis.

These acquired features of the site of interest were then fed into the ProtT5 MLP base model, which consists of an input layer with 1024 dimensions, followed by a first hidden layer with 64 neurons activated by the ReLU activation function. This was followed by a dropout rate of 0.3 and two neurons at the final layer with a SoftMax activation function, utilizing a binary cross-entropy loss function. To prevent overfitting, early stopping and the reduction of the learning rate on the plateau were implemented. Early stopping halts model learning if the validation loss plateaus for a particular number of epochs. Assigned with a patience of five, model learning halts if the validation loss plateaus for five consecutive epochs. Furthermore, the learning rate was reduced during the plateau to aid in model learning. These optimal hyperparameters and models were determined through a 10-fold cross-validation grid search on the training dataset.

#### 2.3.2 Ankh base model

Like the ProtT5 model, we extracted the 87th 1536 feature vectors from the 345 × 1536 embedding matrix for further analysis. This 1536D feature vector of the site of interest was then inputted into the Ankh MLP base model. The Ankh MLP base model comprises an input layer of size 1536, followed by a series of hidden layers. The first hidden layer consists of 512 neurons activated by the ReLU activation function, with a dropout rate of 0.3. Subsequently, this hidden layer is followed by hidden layers with 256 and 32 neurons, respectively, each activated by the ReLU activation function and incorporating a dropout rate of 0.3. The final classification layer comprises of 2 neurons with a SoftMax activation function and utilizes a binary cross-entropy loss function. To mitigate overfitting, techniques such as early stopping with the patience of five and the reduction of the learning rate during the plateau phase were used.

#### 2.3.3 Meta-classifier

**Figure 1.**
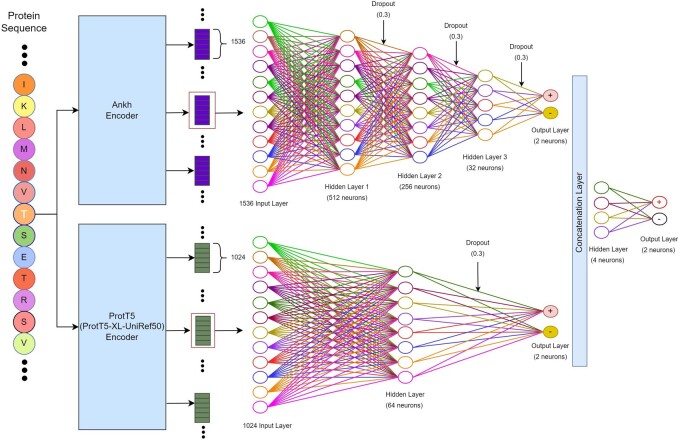
The overall framework of Stack-OglyPred-PLM. Beads with letters represent protein sequences. The large rectangular box represents ProtT5 and Ankh PLM. Small rectangular boxes at top, right are 1024 per residue embedding produced by ProtT5 PLM, and the rectangular boxes below this are 1536 per residue embedding produced by Ankh PLM. Empty circles represent neurons. Each neuron is connected to the next hidden layer’s neurons via links like a biological axon-synapse-dendrite connection. A dropout of 0.3 means 30% of neurons are switched off randomly while training the model.

The meta-classifier integrates the prediction probabilities produced by both the ProtT5 and Ankh MLP base models to make final predictions. Combining the output feature vectors from both models, the MLP-based meta-classifier is trained. This meta-classifier comprises four neurons in the first hidden layer (utilizing the ReLU activation function) and two neurons in the output layer, using a SoftMax activation function. Its function is to discern whether the site is *O-*linked glycosylated. A decision boundary of 0.5 is used in the meta-classifier: if the prediction probability exceeds or equals 0.5, the site is categorized as positive; otherwise, it is regarded as a negative *O-*linked glycosylated site. The hyperparameters used in the Stack-OGlyPred-PLM architecture are outlined in Supplementary Table S1, while Fig. 1 illustrates the comprehensive framework of the Stack-OglyPred-PLM. Moreover, Supplementary Material provides brief information on model evaluation techniques and performance metrics. The different ML and DL models used in this study are briefly explained in the Supplementary Section.

## 3 Results

ProtT5 and Ankh PLMs were used to generate the contextualized embeddings of each amino acid. The full protein sequence of dbPTM (Li *et al.* 2022), NetOGlyc-4.0 (Steentoft *et al.* 2013), and GalNAc-T (Nielsen *et al.* 2022) were fed as an input to those PLMs (Elnaggar *et al.* 2022, 2023). Only “S/T” embeddings were extracted and used as input to the Stack-OglyPred-PLM to train and predict *O-*linked glycosylation sites. Using DeepLoc 2.0 (Thumuluri *et al.* 2022), we separated nucleocytoplasmic proteins from the dbPTM *O-*linked glycosylation database to develop a model focusing on nucleocytoplasmic *O-*linked glycosylation PTM prediction. In addition, DeepLoc 2.0 was used to identify extracellular proteins from the NetOGlyc-4.0 database to create a model specialized for extracellular *O-*linked glycosylation PTM prediction. To validate the results from the stack-generalization extracellular (GalNAc) *O-*linked glycosylation model, we trained and tested the Stack-OglyPred-PLM architecture with the extracellular proteins obtained from the seminal GalNAc-T database (Nielsen *et al.* 2022). The psi-cd-hit tool (threshold of 30%) was applied to eliminate redundant proteins having sequence similarity of 30% or greater while preserving one protein sequence from both the independent testing and training datasets, mitigating the risk of overfitting as well as maintaining diversity (Huang *et al.* 2010). We conducted stratified 10-fold cross-validation with grid search on the dbPTM and NetOGlyc-4.0 training datasets to obtain optimal hyperparameters. Finally, a performance assessment of the nucleocytoplasmic (GlcNAc) model and extracellular (GalNAc) model was conducted on independent test datasets, followed by comparative analysis with the widely used NetOGlyc-4.0 predictor.

### 3.1 Performance of models on the dbPTM nucleocytoplasmic *O-*linked glycosylation datasets

#### 3.1.1 10-Fold cross-validation on the dbPTM nucleocytoplasmic training set with ProtT5 features

The optimal hyperparameters and models were discovered through a stratified 10-fold cross-validation (CV) on the dbPTM nucleocytoplasmic training dataset, and the results are elaborated in Table 3. We used contextualized embeddings extracted from the ProtT5 protein language model, with a feature vector length of 1024, with particular attention given to the “S/T” token. Among the compared models, the MLP architecture exhibited superior performance, achieving a mean MCC, mean ACC, mean SN, and mean SP of 0.455 ± 0.028, 0.727 ± 0.013, 0.701 ± 0.019, and 0.753 ± 0.026, respectively, across the 10-fold CV.

**Table 3.** Results of the 10-fold CV on the dbPTM nucleocytoplasmic training dataset using various models when training datasets are encoded with ProtT5 PLM. The highest value in each column have been highlighted in bold.

Models	MCC ± 1 SD	ACC ± 1 SD	SN ± 1 SD	SP ± 1 SD
LR	0.423 ± 0.026	0.707 ± 0.012	0.604 ± 0.021	0.810 ± 0.022
XGBoost	0.418 ± 0.017	0.708 ± 0.009	0.671 ± 0.013	0.745 ± 0.011
Random forest	0.421 ± 0.026	0.706 ± 0.012	0.604 ± 0.018	0.808 ± 0.017
SVM	0.443 ± 0.025	0.712 ± 0.012	0.571 ± 0.019	**0.853 ± 0.017**
MLP	**0.455 ± 0.028**	**0.727 ± 0.013**	**0.701 ± 0.019**	0.753 ± 0.026

#### 3.1.2 10-Fold cross-validation on the dbPTM nucleocytoplasmic training set with Ankh features

We further explored the effectiveness of the Ankh pre-trained PLM by conducting a stratified 10-fold CV on the dbPTM nucleocytoplasmic training dataset. The results obtained from various models are presented in Table 4. We used contextualized embeddings from Ankh PLM (feature vector length = 1536), specifically focusing on the “S/T” token. The MLP model demonstrated better performance, yielding mean MCC, mean ACC, mean SN, and mean SP of 0.467 ± 0.019, 0.731 ± 0.009, 0.662 ± 0.016, and 0.800 ± 0.012, respectively. These large, pre-trained PLMs display an enhanced ability to capture complex protein patterns, thereby improving accuracy and generalization.

**Table 4.** Results of the 10-fold CV on the dbPTM nucleocytoplasmic training dataset using various models when training datasets are encoded with Ankh PLM. The highest value in each column have been highlighted in bold.

Models	MCC ± 1 SD	ACC ± 1 SD	SN ± 1 SD	SP ± 1 SD
LR	0.437 **±** 0.024	0.717 **±** 0.012	0.668 **±** 0.028	0.766 **±** 0.019
XGBoost	0.405 **±** 0.016	0.701 **±** 0.008	0.662 **±** 0.019	0.741 **±** 0.015
Random forest	0.401 **±** 0.023	0.696 **±** 0.011	0.600 **±** 0.028	0.793 ± 0.022
SVM	0.421 **±** 0.039	0.709 **±** 0.019	0.692 **±** 0.038	0.727 **±** 0.058
MLP	**0.467 ± 0.019**	**0.731 ± 0.009**	**0.662 ± 0.016**	**0.800 ± 0.012**

#### 3.1.3 10-Fold cross-validation on the dbPTM nucleocytoplasmic training set using stacked generalization

We trained separate MLP models with ProtT5 and Ankh PLM embeddings on the dbPTM nucleocytoplasmic *O-*linked glycosylation training dataset, and we combined the prediction scores from these base models to train the MLP meta-classifier. This meta-model learns to integrate predictions from the base models, thereby enhancing generalization and performance compared to individual models. The results of the 10-fold CV demonstrate mean MCC, ACC, SN, and SP of 0.477 ± 0.036, 0.738 ± 0.018, 0.715 ± 0.030, and 0.760 ± 0.026, respectively, surpassing those of the individual base models. As a result, we have selected the stacked generalization model as our primary model for intracellular nucleocytoplasmic *O-*linked glycosylation PTM prediction purposes. The Supplementary Section provides a detailed explanation of the algorithm used to conduct a 10-fold CV during the stacking process (Wolpert 1992).

#### 3.1.4 Testing on dbPTM nucleocytoplasmic *O-*linked glycosylation independent test dataset

To assess the performance of the trained model (Stack-OglyPred-PLM) on an independent dbPTM nucleocytoplasmic test dataset, we trained Stack-OglyPred-PLM on the overall dbPTM nucleocytoplasmic training dataset and tested it using an independent dbPTM nucleocytoplasmic *O-*linked glycosylation test dataset. None of the *O-*linked glycosylation sites (and their corresponding protein sequences) used in Stack-OglyPred-PLM training is present in the independent test dataset. The model achieved MCC, ACC, SN, and SP values of 0.282, 82.3%, 76.2%, and 82.5%, respectively. In addition, Stack-OglyPred-PLM identified 22 320 samples as true negatives (TN), 810 as true positives (TP), 4711 samples as false positives (FP), and 252 as false negatives (FN). Furthermore, Table 5 provides details on the results obtained from the best-performing MLP algorithm, along with the stacked generalization method.

**Table 5.** Performance metrics of various trained models on the dbPTM nucleocytoplasmic independent test set with ProtT5 features. The highest value in each column have been highlighted in bold.

Model	MCC	ACC	SN	SP
MLP (ProtT5)	0.265	0.805	**0.765**	0.806
MLP (Ankh)	0.262	0.815	0.734	0.811
Stack-OglyPred-PLM	**0.282**	**0.823**	0.762	**0.825**

### 3.2 Performance of models on the NetOGlyc-4.0 extracellular *O-*linked glycosylation datasets

#### 3.2.1 10-Fold cross-validation on the NetOGlyc-4.0 extracellular training set with ProtT5 features

To optimize hyperparameters and determine the most suitable ML or DL model for the NetOGlyc-4.0 extracellular dataset, we conducted a stratified 10-fold CV on the NetOGlyc-4.0 extracellular training dataset. The results of this analysis are presented in Table 6. Notably, the MLP architecture demonstrated superior performance, with mean MCC, mean ACC, mean SN, and mean SP of 0.635 ± 0.067, 0.815 ± 0.032, 0.848 ± 0.067, and 0.781 ± 0.050, respectively, across the 10-fold cross-validation.

**Table 6.** Results of the 10-fold CV on the NetOGlyc-4.0 extracellular training dataset using various models when training datasets are encoded with ProtT5 PLM. The highest value in each column have been highlighted in bold.

Models	MCC ± 1 SD	ACC ± 1 SD	SN ± 1 SD	SP ± 1 SD
LR	0.582 ± 0.058	0.790 ± 0.029	0.824 ± 0.040	0.756 ± 0.038
XGBoost	0.609 ± 0.052	0.803 ± 0.026	0.828 ± 0.042	0.778 ± 0.052
Random forest	0.574 ± 0.075	0.785 ± 0.038	0.771 ± 0.077	**0.799 ± 0.061**
SVM	0.623 ± 0.034	0.810 ± 0.018	**0.850 ± 0.030**	0.769 ± 0.043
**MLP**	**0.635 ± 0.067**	**0.815 ± 0.032**	0.848 ± 0.067	0.781 ± 0.050

#### 3.2.2 10-Fold cross-validation on the NetOGlyc-4.0 extracellular training set with Ankh features

We conducted stratified 10-fold cross-validation on the NetOGlyc-4.0 extracellular training dataset utilizing Ankh PLM embeddings. The MLP model demonstrated remarkable performance, achieving mean MCC, mean ACC, mean SN, and mean SP values of 0.632 ± 0.056, 0.814 ± 0.028, 0.828 ± 0.067, and 0.800 ± 0.039, respectively. The outcomes of different ML and DL models are outlined in Table 7.

**Table 7.** Result of the 10-fold CV on the NetOGlyc-4.0 extracellular training dataset using various models when training datasets are encoded with Ankh PLM. The highest value in each column have been highlighted in bold.

Models	MCC ± 1 SD	ACC ± 1 SD	SN ± 1 SD	SP ± 1 SD
LR	0.579 **±** 0.052	0.789 **±** 0.026	0.798 **±** 0.032	0.780 **±** 0.033
XGBoost	0.582 **±** 0.091	0.790 **±** 0.045	0.819 **±** 0.050	0.761 **±** 0.049
Random forest	0.561 **±** 0.069	0.780 **±** 0.034	0.795 **±** 0.049	0.764 ± 0.044
SVM	0.613 **±** 0.080	0.805 **±** 0.040	0.824 **±** 0.051	0.786 **±** 0.047
MLP	**0.632 ± 0.056**	**0.814 ± 0.028**	**0.828 ± 0.067**	**0.800 ± 0.039**

#### 3.2.3 10-Fold cross-validation on the NetOGlyc-4.0 extracellular training set with stacked generalization

We trained an MLP model with ProtT5 embeddings and another MLP model with Ankh embeddings on the NetOGlyc-4.0 extracellular dataset. Subsequently, we utilized the prediction probabilities of these base models to train a meta-MLP classifier. The 10-fold cross-validation results produced by the stacked generalization model were 0.640 ± 0.062 for MCC, 0.818 ± 0.032 for ACC, 0.855 ± 0.043 for SN, and 0.780 ± 0.069 for SP on the NetOGlyc-4.0 training dataset. The outcomes achieved through this stacking algorithm surpassed those of the individual base models. Moreover, we performed 10-fold CV on NetOGlyc-4.0 and dbPTM training datasets, holding out one-fold for independent testing with distinct proteins and sites, and the results are presented in the Supplementary “10-fold CV that simulates independent testing” Section.

#### 3.2.4 Testing on the NetOGlyc-4.0 extracellular *O-*linked glycosylation independent test dataset

The Stack-OglyPred-PLM, which was trained on NetOGlyc-4.0 training dataset, was evaluated using the NetOGlyc-4.0 extracellular independent test dataset. The model yielded MCC, ACC, SN, and SP values of 0.464, 89.7%, 90.5%, and 89.6%, respectively. In addition, the model accurately classified 1781 instances as true negatives (TN) and 77 instances as true positives (TP), while incorrectly classifying 205 instances as false positives (FP) and 8 instances as false negatives (FN). Supplementary Table S3 provides a detailed overview of the outcomes derived from using each MLP with its respective ProtT5 and Ankh PLM embeddings, in addition to the stacked generalization method. Moreover, Supplementary Table S3 clearly illustrates that the stacked generalization method surpasses the results achieved by the individual base models. Results from the GalNAc-T extracellular *O-*linked glycosylation dataset (Nielsen *et al.* 2022) are detailed in Supplementary Materials. These results confirm the model’s effectiveness, as they match findings from the NetOGlyc-4.0 extracellular test dataset.

### 3.3 ROC curve plots using the NetOGlyc-4.0 extracellular training dataset

**Figure 2.**
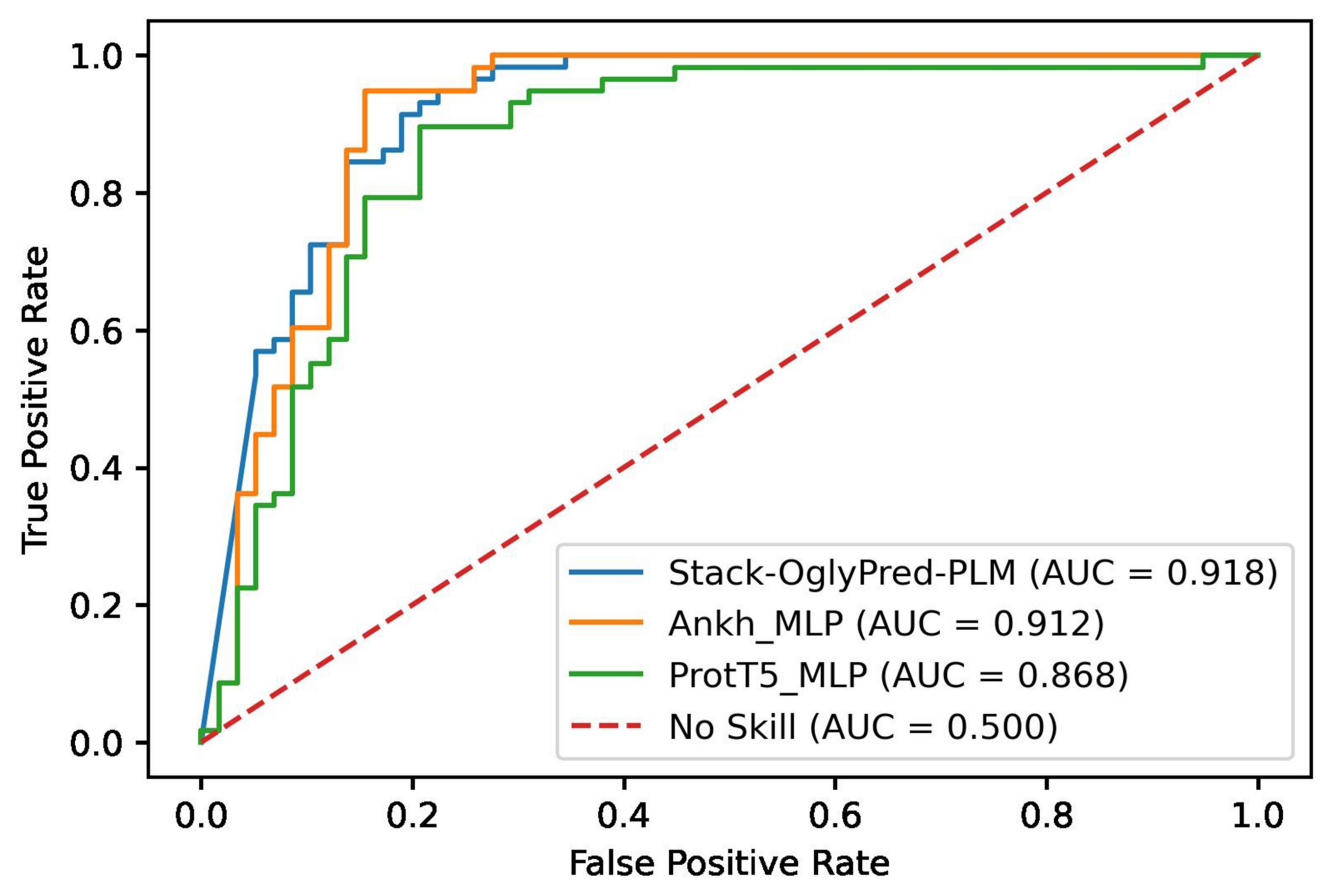
ROC curve obtained from ProtT5; Ankh-based MLP along with Stack-OglyPred-PLM on 10% training dataset split from the extracellular training dataset.

To assess the efficacy of Stack-OglyPred-PLM in comparison to individual base models, ProtT5 and Ankh PLM-based MLPs, we generated receiver operating characteristic (ROC) and precision-recall (PR) curves using a 10% test subset separated from the NetOGlyc-4.0 Extracellular Training set. Figure 2 illustrates that the ROC curve of Stack-OglyPred-PLM is better than that of ProtT5 and Ankh-based MLPs. Similarly, Supplementary Fig. S1 demonstrates that the precision-recall curve of Stack-OglyPred-PLM surpasses those of the individual base models, indicating the superiority of the stacked approach over individual base models.

### 3.4 *t*-Distributed stochastic neighbor embedding plot

**Figure 3.**
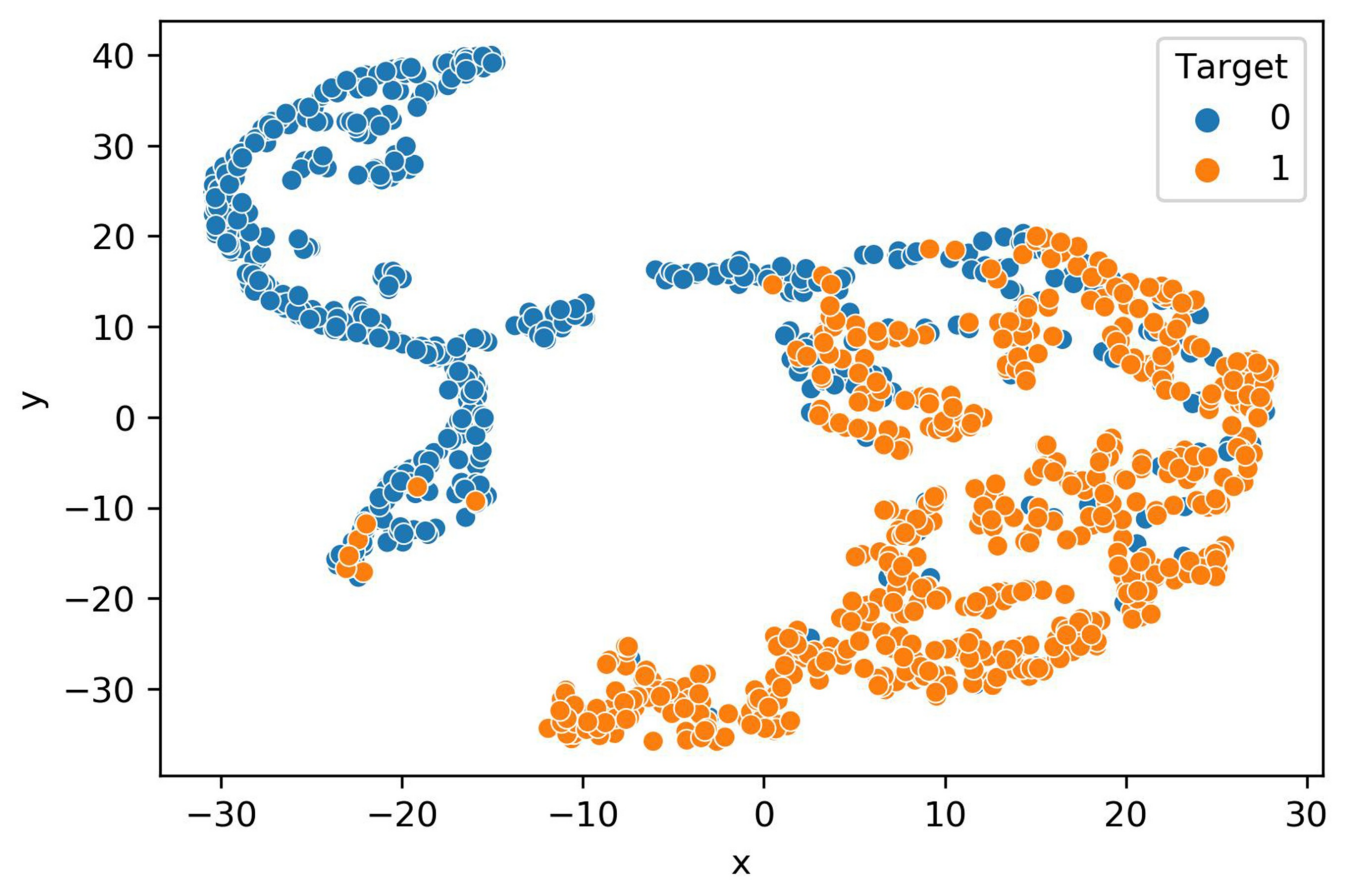
t-SNE visualization of the acquired features. Learned features from the trained Stacked model (Stack-OglyPred-PLM).

The t-SNE technique was utilized to evaluate the classification efficacy of embeddings derived from the penultimate dense layer of the Stack-OglyPred-PLM model (Maaten and Hinton 2008). This method facilitates the projection of these features into a 2D space to delineate class boundaries. A learning rate of 50 was used for t-SNE to visualize the scatter plot of ProtT5 features and feature vectors obtained from the second to last fully connected (FC) layer of the trained ProtT5 MLP, Ankh MLP, and Stack-OglyPred-PLM framework. The plots were generated using randomly selected NetOGlyc-4.0 training data points, evenly distributed between 582 negative and 582 positive samples. Figure 3 demonstrates positive samples (points on the right side), and negative samples (points on the left side) are distinctly clustered, indicating that the Stack-OglyPred-PLM is proficient in learning extracellular *O-*linked glycosylation patterns and thus capable of accurately classifying negative and positive samples in a 2D space. Supplementary Figure S3A shows clusters of positive and negative samples from ProtT5 PLM, but the class boundaries between them are not well-defined. In contrast, Supplementary Fig. S3B and C display the t-SNE plots of features from the penultimate FC layer the trained ProtT5 MLP and Ankh MLP, respectively, where positive and negative sites are separated into distinct clusters.

### 3.5 Comparison of Stack-OglyPred-PLM with other *O-*linked glycosylation site predictors

To evaluate Stack-OglyPred-PLM’s performance against the NetOGlyc-4.0 predictor, we trained our model using the NetOGlyc-4.0 training dataset and assessed its performance on the NetOGlyc-4.0 independent test dataset. Our stacking approach yielded MCC, ACC, SN, and SP values of 0.464, 89.7%, 90.5%, and 89.6%, respectively. Stack-OglyPred-PLM yielded 77 as true positive (TP), 1781 as true negative (TN), 205 as false positive (FP), and 8 as false negative (FN). Comparatively, when we submitted 17 independent proteins along with their positive and negative sites to the NetOGlyc-4.0 predictor, it produced MCC, ACC, SN, and SP scores of 0.240, 69.0%, 89.4%, and 68.1%, respectively.

To compare our method with other predictors like OGP and Captor, we excluded all *O-*linked and non-*O-*linked glycosylation sites present in the Captor independent test dataset from the OGP training dataset. We then trained our model on this training dataset and tested it on the Captor-independent test dataset. As shown in Table 8, Stack-OglyPred-PLM outperformed both Captor and OGP in detecting *O*-linked glycosylation, with better positive detection capabilities. Specifically, OglyPred-PLM correctly identified 1096 samples as TN and 252 samples as TP, while it misclassified 211 samples as FP and 88 samples as FN. Notably, OglyPred-PLM, Captor, and OGP were all calibrated and evaluated using the same experimentally verified OGP dataset.

**Table 8.** Prediction performance of stack-OglyPred-PLM compared to Captor and OGP *O-*linked glycosylation predictors on the Captor independent test set. The highest value in each column have been highlighted in bold.

Methods	MCC	ACC	SN	SP
Stack-OglyPred-PLM	**0.521**	**0.818**	**0.741**	**0.838**
Captor	0.278	0.795	0.619	0.798
OGP	0.160	0.780	0.485	0.796

Moreover, to evaluate the performance of Stack-OglyPred-PLM compared to other established predictors, we trained our model using the NetOGlyc-4.0 training dataset, which excludes both the NetOGlyc-4.0 independent test dataset and the SPRINT-Gly independent test dataset. In addition, to mitigate potential bias, we applied an under-sampling technique on the training dataset with a positive-to-negative sample ratio of 1:2. The evaluation was conducted without an under-sampled SPRINT-Gly independent test dataset. The model achieved MCC, ACC, SN, SP, and PRE scores of 0.240, 83.7%, 77.2%, and 83.5%, respectively (please refer to Table 9). These results surpass those obtained from the SPRING-Gly method. Notably, Stack-OglyPred-PLM showed a decent positive detection capability for *O-*linked glycosylation (77.2%), significantly higher than SPRINT-GLY’s 32.9%. The confusion matrix for the MLP architecture reveals that the model correctly identified 2831 samples as TN and 61 as TP but misclassified 545 samples as FP and 18 as FN. Results for other consensus-based models, GPP, GlycoMine, and GlycoPP, were adopted from SPRINT-Gly. This outcome demonstrates the superiority of our predictor over the other quintessential predictors.

**Table 9.** Prediction performance of Stack-OglyPred-PLM compared to SPRINT-Gly and other *O-*linked glycosylation predictors on the SPRINT-Gly independent test set. The highest value in each column have been highlighted in bold.

Methods	MCC	ACC	SN	SP
Stack-OglyPred-PLM	**0.240**	0.837	**0.772**	**0.838**
SPRINT-Gly	0.191	0.940	0.329	0.954
Consensus-based model	0.042	0.227	0.223	0.216
GPP	0.047	0.489	0.671	0.216
GlycoMine	0.100	**0.978**	0.027	0.999
GlycoPP	0.061	0.973	0.038	0.994

## 4 Conclusion

We developed a stacked-generalization approach that utilizes prediction probabilities from two MLP-based base models. These base models are built on ProtT5 and Ankh embeddings, respectively. The prediction scores from these base models are used to train a meta-MLP model that categorizes extracellular *O-*linked glycosylation sites. A similar model was developed to classify nucleocytoplasmic *O-*linked glycosylation sites. Our method was tested on a newly curated extracellular *O-*linked glycosylation test set derived from the NetOGlyc-4.0 dataset, achieving an MCC of 0.464, ACC of 89.7%, SN of 90.5%, and SP of 87.9%. For the nucleocytoplasmic *O*-linked glycosylation dataset from the dbPTM database, our predictor achieved an MCC of 0.282, ACC of 82.3%, SN of 76.2%, and SP of 82.5%.

The improved performance of Stack-OglyPred-PLM can be attributed to the site-specific embeddings generated from ProtT5 and Ankh PLMs. Furthermore, we have created an accessible benchmark dataset for human nucleocytoplasmic and extracellular *O-*linked glycosylation sites. The extracellular *O-*linked glycosylation dataset includes glycoproteins and their sites from the NetOGlyc-4.0 and GalNAc-T datasets, while the nucleocytoplasmic dataset includes glycoproteins and sites from the dbPTM dataset. This effort aims to enhance reproducibility and enable equitable comparisons among different methods for predicting *O-*linked glycosylation sites. By providing these resources, we seek to establish a uniform evaluation framework for diverse methodologies targeting the prediction of *O-*linked glycosylation sites.

## Supplementary Material

btae643_Supplementary_Data
