# Supplementary material for: Prediction of human O-linked glycosylation sites using stacked generalization and embeddings from pre-trained protein language model
Source: Bioinformatics. 2024 Oct 24;40(11):btae643. doi: 10.1093/bioinformatics/btae643 (PMC11552629; doi:10.1093/bioinformatics/btae643)
Supplement: btae643_Supplementary_Data [file btae643_supplementary_data.zip › Supplementary_materials.docx]

Prediction of human *O-*linked glycosylation sites using stacked generalization and embeddings from pre-trained protein language model

Subash Chandra Pakhrin^1, *^, Neha Chauhan^2^, Salman Khan^3^, Jamie Upadhyaya^1^, Moriah Rene Beck^4^ and Eduardo Blanco^5^

^1^Department of Computer Science and Engineering Technology, University of Houston-Downtown, 1 Main St., Houston, Texas 77002, USA, ^2^School of Computing, Wichita State University, 1845 Fairmount St., Wichita, Kansas 67260, USA, ^3^Department of Computer Science, The University of Texas at Austin, 2317 Speedway, GDC 2.302 Austin, Texas 78712, USA, ^4^Department of Chemistry and Biochemistry, Wichita State University, 1845 Fairmount St., Wichita, Kansas 67260, USA, ^5^Department of Computer Science, University of Arizona, 1040 4^th^ St., Tucson, Arizona 85721, USA

*To whom correspondence should be addressed.

Tel: +1 713 221 5819; Fax: +1 713 223 7407; pakhrins@uhd.edu

**Stacked generalization algorithm**

Stacked generalization is an ensemble technique designed to improve the predictive performance of base models by combining their output predictions. This method involves concatenating the predictions of each base model into a feature vector, which is then used to train a meta-classifier. However, there's a risk of overfitting the meta-classifier if any base model overfits the training data. To address this issue, K-fold cross-validation is employed.

The first step in stacked generalization is to create a training set and a separate test set. This is accomplished through K-fold cross-validation, where the dataset is divided into K equal folds. In each iteration, one-fold is used as the test set, while the remaining folds are utilized as the training set for all base models. The predictions of the trained models on the test set are then combined into a feature vector for training the meta-classifier. After K iterations, each base model produces predictions that are combined with the corresponding ground truth labels to train the meta-classifier. This results in a trained meta-classifier.

Next, all base models are retrained using the entire dataset, leading to trained base models. Following this procedure, an independent test dataset is employed to further evaluate the stacked generalization model.

**Machine learning and deep learning models**

**Support Vector Machine (SVM)** is a supervised learning algorithm employed for classification and regression tasks, aiming to discover an optimal hyperplane for segregating data into distinct classes. In instances where data isn't linearly separable, SVM utilizes the kernel trick to map it into a higher-dimensional space, enhancing separability.

**Random Forest (RF)** serves as a prevalent ensemble learning approach in machine learning, extending decision trees (DT) by amalgamating multiple DT to generate predictions. For classification, it aggregates individual tree predictions through majority voting, culminating in the final prediction based on the class with the highest votes.

**Logistic Regression (LR)** stands as a machine learning technique proficient in binary classification, estimating the probability of an instance belonging to a specific class by fitting a logistic (sigmoid) function to the input features, thereby crafting a linear decision boundary that partitions the two classes.

**Extreme Gradient Boosting (XGBoost)** belongs to the gradient boosting methodology, which involves iteratively appending weak models (decision trees) to rectify previous model errors. It optimizes a designated loss function, progressively refining the model by iteratively identifying the best-fitting model components.

**Multi-Layer Perceptron (MLP)** represents a feedforward artificial neural network extensively applied in classification and regression endeavors. Comprising multiple layers of interconnected neurons, each neuron conducts a weighted summation of inputs and then applies a non-linear activation function to yield an output.

**Model evaluation and performance metrics**

In this investigation, a 10-fold cross-validation approach was employed to assess the model's performance and ascertain its resilience and applicability. During the 10-fold cross-validation process, the training dataset was divided into ten equal segments. Subsequently, one segment was set aside for validation purposes while training was conducted on the remaining nine segments, with this procedure iterated until all segments were utilized for validation. Unless explicitly stated otherwise, the outcomes of the 10-fold cross-validation are presented as the mean value with a ± 1 standard deviation.

To gauge the effectiveness of each model, accuracy (ACC), sensitivity (SN), specificity (SP), and Matthew’s correlation coefficient (MCC) were utilized. ACC quantifies the correctly predicted residues relative to the total residues (Equation (1)), SN assesses the model's capability to discern positive residues (Equation (2)), and SP evaluates the model's accuracy in identifying negative residues (Equation (3)). Meanwhile, MCC offers a comprehensive measure of the model's predictive prowess concerning both positive and negative residues (Equation (4)).

$$Accuracy= \frac{TP+TN}{TP+TN+FP+TN} \times100 (1)$$

$$Sensitivity= \frac{TP}{TP+FN} \times100 (2)$$

$$Specificity= \frac{TN}{TN+FP} \times100 (3)$$

$$MCC= \frac{\left( TP \right)\left( TN \right)-\left( FP \right)(FN)}{\sqrt{\left( TP+FP \right)\left( TP+FN \right)\left( TN+FP \right)\left( TN+FN \right)}} (4)$$

**Supplementary Table 1.** Hyperparameters of Stack-OglyPred-PLM

| Model | Layer | Hyperparameters | | |
| --- | --- | --- | --- | --- |
|  |  | Activation Function | Size | Drop-Out |
| ProT5 | Dense | ReLU | 64 | 0.3 |
|  |  | SoftMax | 2 | - |
| Ankh | Dense | ReLU | 512 | 0.3 |
|  |  | ReLU | 256 | 0.3 |
|  |  | ReLU | 32 | 0.3 |
|  |  | SoftMax | 2 | - |
| Meta | Dense | ReLU | 4 | - |
|  |  | SoftMax | 2 | - |

**GalNAc-T dataset**

To validate the findings obtained from the NetOGlyc-4.0 dataset, we retrieved 830 glycoproteins from pioneering research conducted by Nielsen et al (Nielsen, et al., 2022). To focus our model exclusively on the extracellular space, we subjected all 830 proteins obtained from Nielsen et al.'s dataset to DeepLoc 2.0 subcellular localization software (Thumuluri, et al., 2022). The DeepLoc 2.0 software identified 167 proteins as extracellular.

Furthermore, to eliminate redundant proteins and maintain diversity, we applied the psi-cd-hit algorithm with a 30% threshold (Huang, et al., 2010). The filtering process reduced the number of proteins from 167 to 150. Subsequently, we divided these 150 proteins into 135 for training and 15 for independent testing.

From the 135 training proteins, we extracted 525 positive "S/T" extracellular *O-*linked glycosylation sites and 16,887 negative extracellular *O-*linked glycosylation sites, excluding the positives. To address the imbalance issue in the training dataset, we randomly under-sampled the 16,887 negative sites to equalize their number with the positives.

Additionally, we identified 84 extracellular positive *O-*linked glycosylation sites and 1,382 negative extracellular *O-*linked glycosylation sites, excluding the positives from the 15 independent testing proteins. Detailed statistics regarding the number of training and testing sites are presented in Supplementary Table 2.

**Supplementary Table 2.** Positive and Negative extracellular O-linked Glycosylation sites for Training and Independent Testing (derived from Nielsen et al GalNAc-T dataset).

| **Data set** | **Number of Proteins** | **Positive** | **Negative** |
| --- | --- | --- | --- |
| Training | 135 | 525 | 525 |
| Independent Test | 15 | 84 | 1,382 |

**Per-Residue Contextualized Embeddings from Ankh**


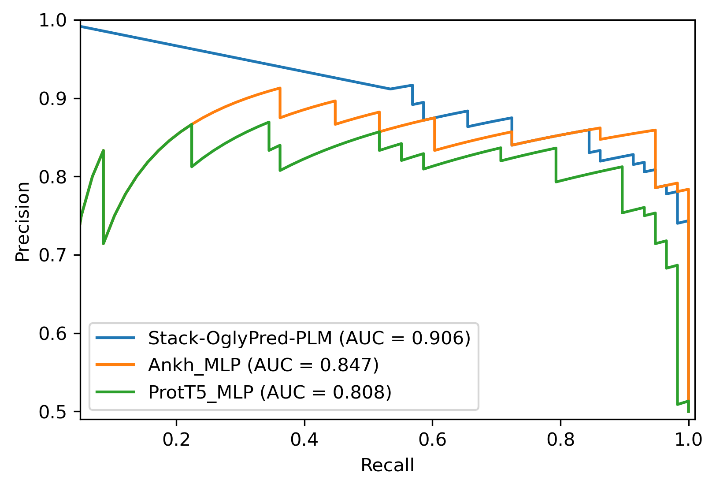
We utilized the complementary Ankh PLM (Elnaggar, et al., 2023) to generate contextualized embeddings for each amino acid. The Ankh model comes in two flavors: the Ankh large model and the Ankh base model. Our research findings indicate that the Ankh large model outperformed the Ankh base model, demonstrating its superior ability to encode intrinsic protein information. Consequently, we opted to employ the features obtained from the Ankh large model for encoding the amino acids of the sequences. Notably, a key distinction from the ProtT5 model is that the Ankh model generated a per-residue contextualized embedding feature vector of length 1536, in contrast to the 1024 feature vector size produced by ProtT5 PLM.

**Supplementary Figure 1.** PR Curve obtained from ProtT5; Ankh-based MLP along with Stack-OglyPred-PLM on 10 % training dataset split from the extracellular training dataset.

**Supplementary Table 3.** Performance metrices of various models on the NetOGlyc-4.0 extracellular independent test dataset.

| **Models** | **MCC** | **ACC** | **SN** | **SP** |
| --- | --- | --- | --- | --- |
| MLP (ProtT5) | 0.427 | 0.880 | 0.894 | 0.879 |
| MLP (Ankh) | 0.394 | 0.862 | 0.882 | 0.862 |
| Stack-OglyPred-PLM | **0.464** | **0.897** | **0.905** | **0.896** |

**Testing on the GalNAc-T extracellular O-linked glycosylation independent test dataset with the stacked generalization model**

To ensure the robustness of the extracellular stack-generalized NetOGlyc4.0 model, we trained this model using the seminal GalNAc-T extracellular O-linked glycosylation dataset (Nielsen et al., 2022). Subsequently, we evaluated the model's performance on the GalNAc-T independent test dataset. The Stack-OglyPred-PLM achieved MCC, ACC, SN, and SP values of 0.486, 0.887, 0.869, and 0.888, respectively, on the independent GalNAc-T extracellular test dataset. These results align with those obtained from the NetOGlyc-4.0 extracellular independent test dataset, further validating the model's effectiveness. Moreover, Supplementary Figure 2 provides an in-depth analysis of the outcomes generated by ProtT5-based MLP, Ankh-based MLP, and StackOglyPred-PLM concerning the NetOGlyc-4.0 extracellular, GalNAc-T extracellular, and dbPTM nucleocytoplasmic independent test datasets.


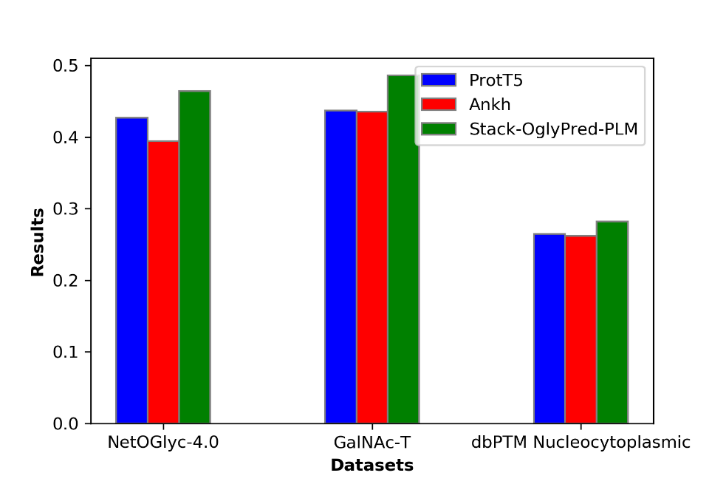


**Supplementary Figure 2.** Results obtained through ProtT5, Ankh, Stacked Generalization method on the NetOGlyc-4.0 extracellular, GalNAc-T extracellular, and dbPTM Nucleocytoplasmic independent test dataset.


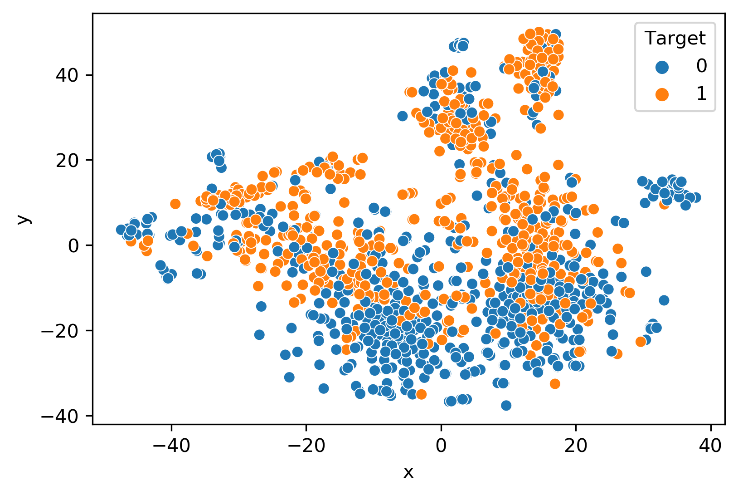


**Supplementary Figure 3A.** t-SNE visualization of the ProtT5 features


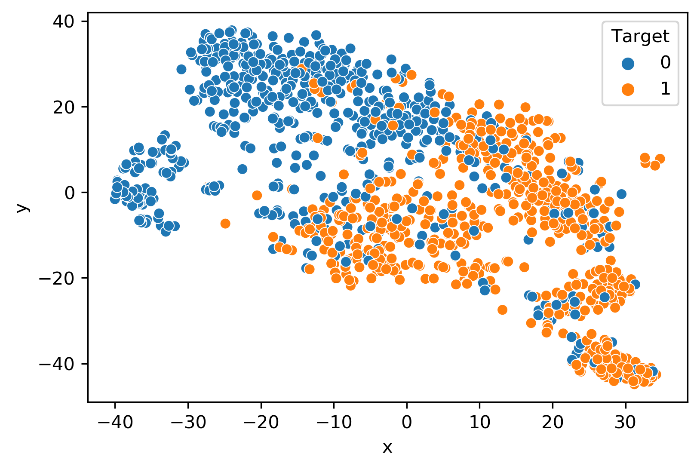


**Supplementary Figure 3B.** t-SNE visualization of the features from penultimate dense layer of trained ProtT5 based MLP model.

**
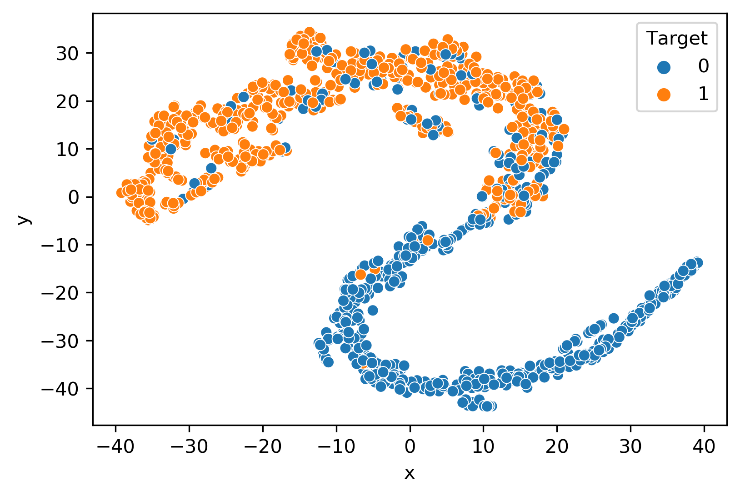
**

**Supplementary Figure 3C.** t-SNE visualization of the features from penultimate dense layer of trained Ankh based MLP model.

**The importance of balanced training**

To support the importance of balancing the training dataset, we conducted an experiments, with results provided in Supplementary Table 4. The findings indicate that when both the training and independent test datasets follow a natural distribution, the model fails to learn effectively. This is because the model predominantly learns about the majority class (negative) and ignores the minority class (positive). The results demonstrate that balanced training is the most effective approach.

Supplementary Table 4: Results obtained from an MLP (ProtT5 embeddings) trained with different positive-to-negative ratios in the OGalNAc extracellular training dataset and evaluated using an independent OGalNAc test dataset.

|  |
| --- |

| Training Dataset (Positive to Negative ratio) | Independent Test Dataset | MCC | ACC | SN | SP |
| --- | --- | --- | --- | --- | --- |
| Equal number of Positive and Negative samples | Positive 85 Negative 1,986 | 0.42 | 87.40% | 91.76% | 87.21% |
| 582 Positive, 1164 negative (1:2) | Positive 85 Negative 1,986 | 0.41 | 89.18% | 81.18% | 89.54% |
| 582 Positive, 1746 negative (1:3) | Positive 85 Negative 1,986 | 0.41 | 90.68% | 74.17% | 91.38% |
| 582 Positive, 2328 negative (1:4) | Positive 85 Negative 1,986 | 0.41 | 92.17% | 68.23% | 93.20% |
| 582 Positive, 2910 negative (1:5) | Positive 85 Negative 1,986 | 0.4 | 93.23% | 60.00% | 94.66% |
| 582 Positive, 3492 negatives (1:6) | Positive 85 Negative 1,986 | 0.4 | 94.01% | 54.11% | 95.72% |
| 582 Positive, 4074 negatives (1:7) | Positive 85 Negative 1,986 | 0.39 | 94.25% | 49.41% | 96.17% |
| 582 Positive, 4656 negatives (1:8) | Positive 85 Negative 1,986 | 0.38 | 95.46% | 37.64% | 97.93% |
| 582 Positive, 5238 negatives (1:9 ratio) | Positive 85 Negative 1,986 | 0.36 | 96.28% | 23.52% | 99.39% |
| Natural Distribution | Positive 85 Negative 1,986 | 0 | 95.89% | 0.00% | 1.00% |

**10 Fold cross-validation that simulates independent testing**

We performed 10-fold cross-validation, using 9 folds of the training data to train the models and 1-fold for independent testing, ensuring that the proteins and positive and negative sites in the independent testing set differed from those in the training folds (there are no overlaps within and across the training and independent test dataset). This process was repeated 10 times. The results for the ProtT5 contextualized embedding for OGalNAc (Extracellular) and the Ankh contextualized embedding for OGalNAc (Extracellular) are shown in Supplementary Table 5 and Supplementary Table 6, respectively. Additionally, the stack generalization model achieved scores of 0.569 ± 0.083 (MCC), 0.783 ± 0.033 (SN), 0.783 ± 0.072 (SP), and 0.783 ± 0.042 (ACC) on the NetOGlyc-4.0 (Extracellular) training dataset, with one-fold consistently held out for independent testing (for 10-fold CV).

Supplementary Table 5. Results of the 10-fold cross-validation on the NetOGlyc-4.0 extracellular training dataset using various models, where the training datasets are encoded with ProtT5 PLM and the training dataset (9 folds), independent dataset (1-fold) separated from training dataset comprises different proteins, along with distinct positive and negative sites from proteins that are not present in the training fold. This process is repeated for 9 additional times.

| Model | MCC | SN | SP | ACC |
| --- | --- | --- | --- | --- |
| MLP | 0.537 ± 0.092 | 0.742 ± 0.124 | 0.787 ± 0.059 | 0.764 ± 0.046 |
| LR | 0.498 ± 0.119 | 0.741 ± 0.114 | 0.751 ± 0.093 | 0.746 ± 0.062 |
| RF | 0.497 ± 0.099 | 0.727 ± 0.074 | 0.772 ± 0.049 | 0.748 ± 0.049 |
| XGBoost | 0.516 ± 0.075 | 0.734 ± 0.100 | 0.774 ± 0.075 | 0.754 ± 0.036 |
| SVM | 0.480 ± 0.181 | 0.646 ± 0.163 | 0.824 ± 0.073 | 0.735 ± 0.092 |

Supplementary Table 6. Results of the 10-fold cross-validation on the NetOGlyc-4.0 extracellular training dataset using various models, where the training datasets are encoded with Ankh PLM and the training dataset (9 folds), independent dataset (1-fold) separated from training dataset comprises different proteins, along with distinct positive and negative sites from proteins that are not present in the training fold. This process is repeated for 9 additional times.

| Model | MCC | SN | SP | ACC |
| --- | --- | --- | --- | --- |
| MLP | 0.544 ± 0.118 | 0.768 ± 0.092 | 0.773 ± 0.068 | 0.770 ± 0.059 |
| LR | 0.529 ± 0.178 | 0.756 ± 0.112 | 0.767 ± 0.109 | 0.762 ± 0.088 |
| RF | 0.503 ± 0.131 | 0.741 ± 0.076 | 0.757 ± 0.102 | 0.749 ± 0.064 |
| XGBoost | 0.500 ± 0.133 | 0.706 ± 0.107 | 0.788 ± 0.067 | 0.747 ± 0.065 |
| SVM | 0.537 ± 0.122 | 0.720 ± 0.123 | 0.810 ± 0.038 | 0.765 ± 0.062 |

We similarly conducted 10-fold cross-validation on the nucleocytoplasmic O-linked glycosylation (GlcNAc) training dataset, using 9 folds for model training and 1-fold for independent testing. This process was repeated 10 times, ensuring that the proteins and both positive and negative sites in the independent fold were distinct from those in the training folds. The results for the ProtT5 contextualized embedding for GlcNAc (nucleocytoplasmic) and the Ankh contextualized embedding for GlcNAc (nucleocytoplasmic) are shown in Supplementary Table 7 and Supplementary Table 8, respectively. Additionally, the stack generalized model achieved scores of 0.430 ± 0.039 (MCC), 0.663 ± 0.030 (SN), 0.763 ± 0.039 (SP), and 0.713 ± 0.018 (ACC) on the dbPTM nucleocytoplasmic training dataset, with one-fold consistently held out for independent testing (where the one-fold test dataset is different from nine-fold training dataset) (for 10-fold CV).

Supplementary Table 7. Results of the 10-fold cross-validation on the dbPTM nucleocytoplasmic training dataset using various models, where the training datasets are encoded with ProtT5 PLM and the training dataset (9 folds), independent dataset (1-fold) separated from training dataset comprises different proteins, along with distinct positive and negative sites from proteins that are not present in the training fold. This process is repeated for 9 additional times.

| Model | MCC | SN | SP | ACC |
| --- | --- | --- | --- | --- |
| MLP | 0.423 ± 0.039 | 0.658 ± 0.062 | 0.760 ± 0.028 | 0.709 ± 0.021 |
| LR | 0.407 ± 0.055 | 0.661 ± 0.070 | 0.742 ± 0.025 | 0.702 ± 0.028 |
| RF | 0.409 ± 0.055 | 0.573 ± 0.058 | 0.822 ± 0.028 | 0.697 ± 0.028 |
| XGBoost | 0.391 ± 0.044 | 0.639 ± 0.047 | 0.749 ± 0.015 | 0.694 ± 0.023 |
| SVM | 0.389 ± 0.030 | 0.634 ± 0.037 | 0.752 ± 0.023 | 0.693 ± 0.015 |

Supplementary Table 8. Results of the 10-fold cross-validation on the dbPTM nucleocytoplasmic training dataset using various models, where the training datasets are encoded with Ankh PLM and the training dataset (9 folds), independent dataset (1-fold) separated from training dataset comprises different proteins, along with distinct positive and negative sites from proteins that are not present in the training fold. This process is repeated for 9 additional times.

| Model | MCC | SN | SP | ACC |
| --- | --- | --- | --- | --- |
| MLP | 0.410 ± 0.078 | 0.636 ± 0.056 | 0.767 ± 0.075 | 0.701 ± 0.038 |
| LR | 0.405 ± 0.067 | 0.636 ± 0.065 | 0.764 ± 0.020 | 0.700 ± 0.034 |
| RF | 0.391 ± 0.061 | 0.591 ± 0.051 | 0.792 ± 0.032 | 0.691 ± 0.031 |
| XGBoost | 0.386 ± 0.041 | 0.636 ± 0.054 | 0.746 ± 0.032 | 0.691 ± 0.021 |
| SVM | 0.392 ± 0.037 | 0.647 ± 0.058 | 0.742 ± 0.036 | 0.694 ± 0.019 |
