# Supplementary figures and images for: Prediction of human O-linked glycosylation sites using stacked generalization and embeddings from pre-trained protein language model
Source: Bioinformatics. 2024 Oct 24;40(11):btae643. doi: 10.1093/bioinformatics/btae643 (PMC11552629; doi:10.1093/bioinformatics/btae643)

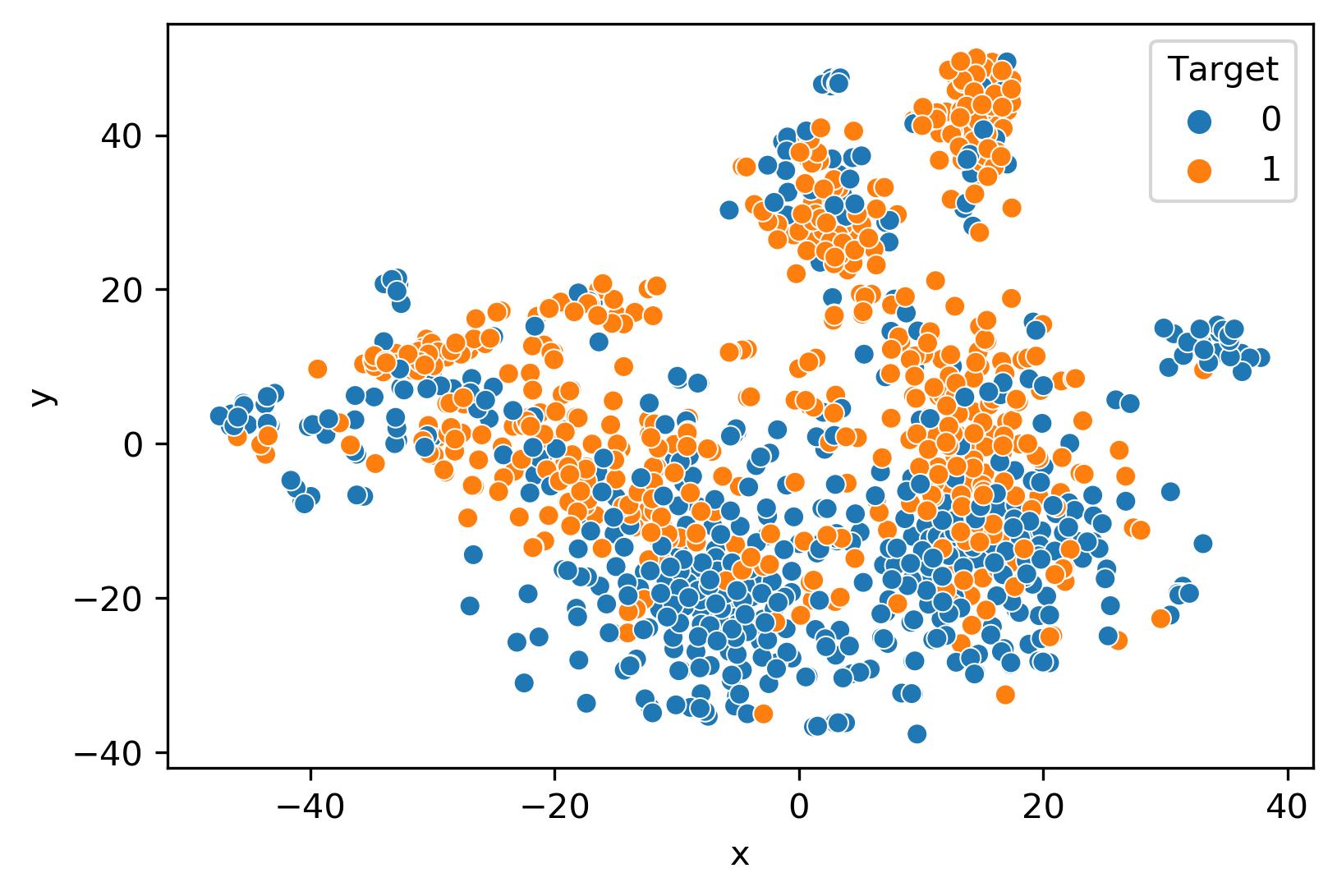

Supplement: btae643_Supplementary_Data [file btae643_supplementary_data.zip › Supplementary_Figure_3A_1200_dpi.jpg]

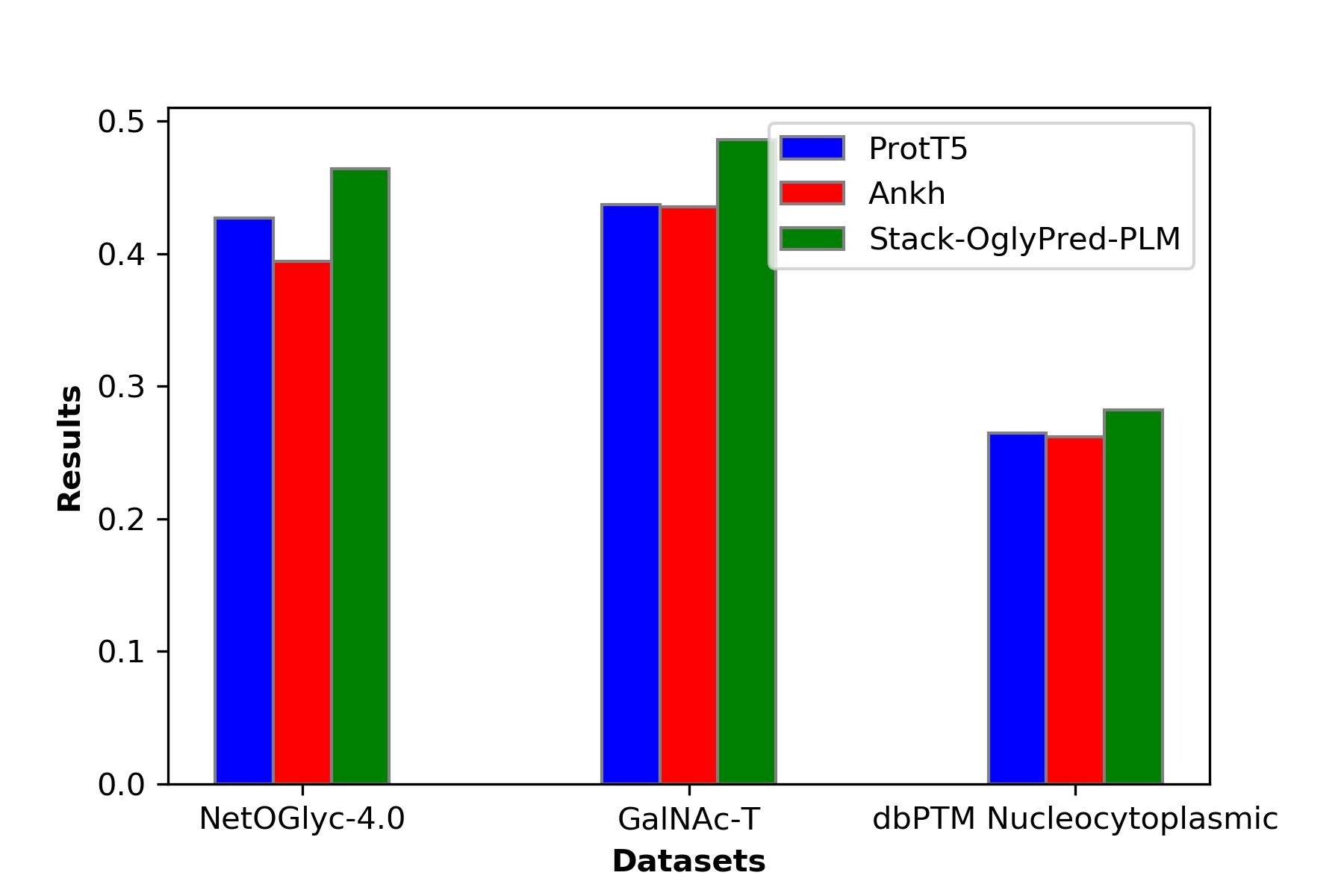

Supplement: btae643_Supplementary_Data [file btae643_supplementary_data.zip › Supplementary_Figure_2_1200_dpi.jpg]

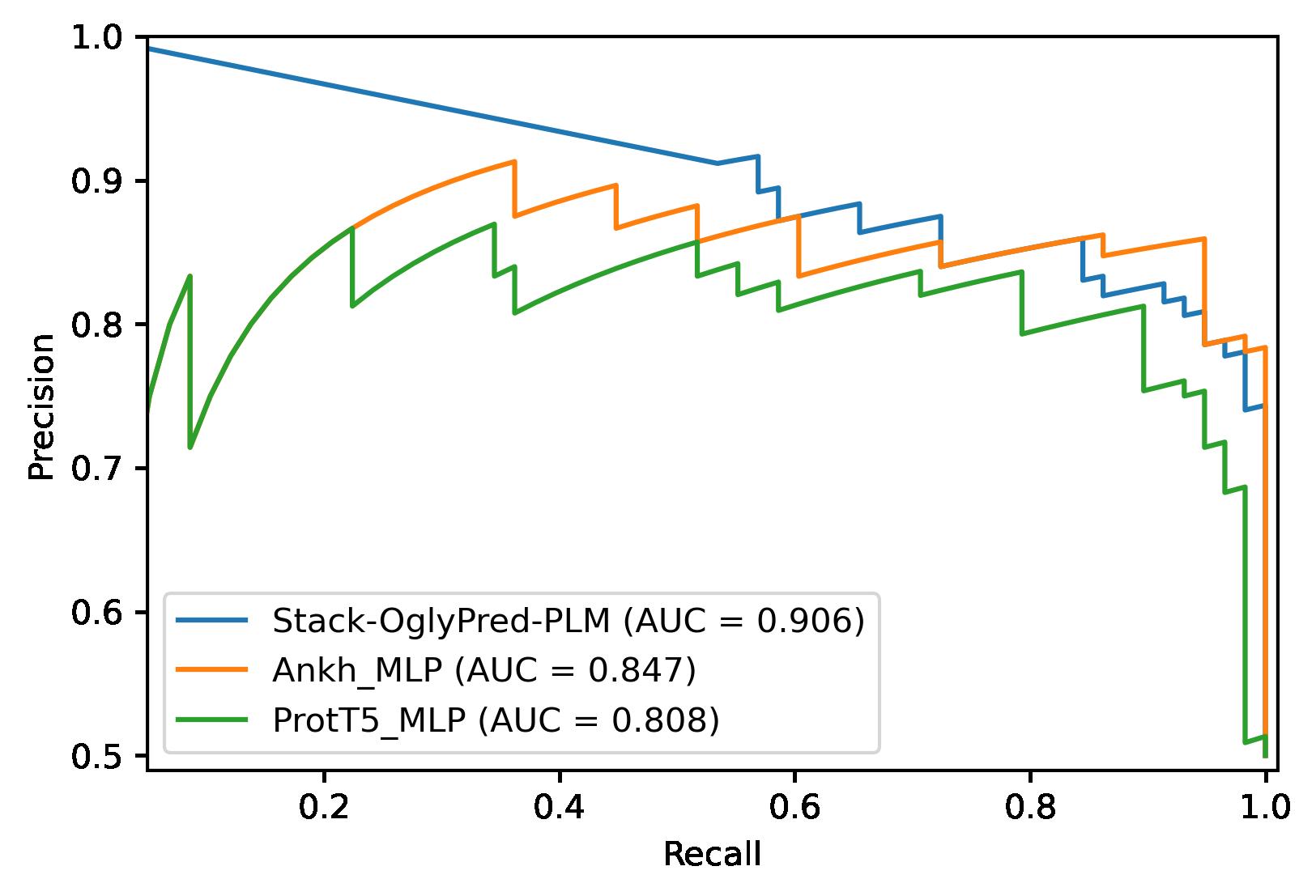

Supplement: btae643_Supplementary_Data [file btae643_supplementary_data.zip › Supplementary_Figure_1_1200_dpi.jpg]

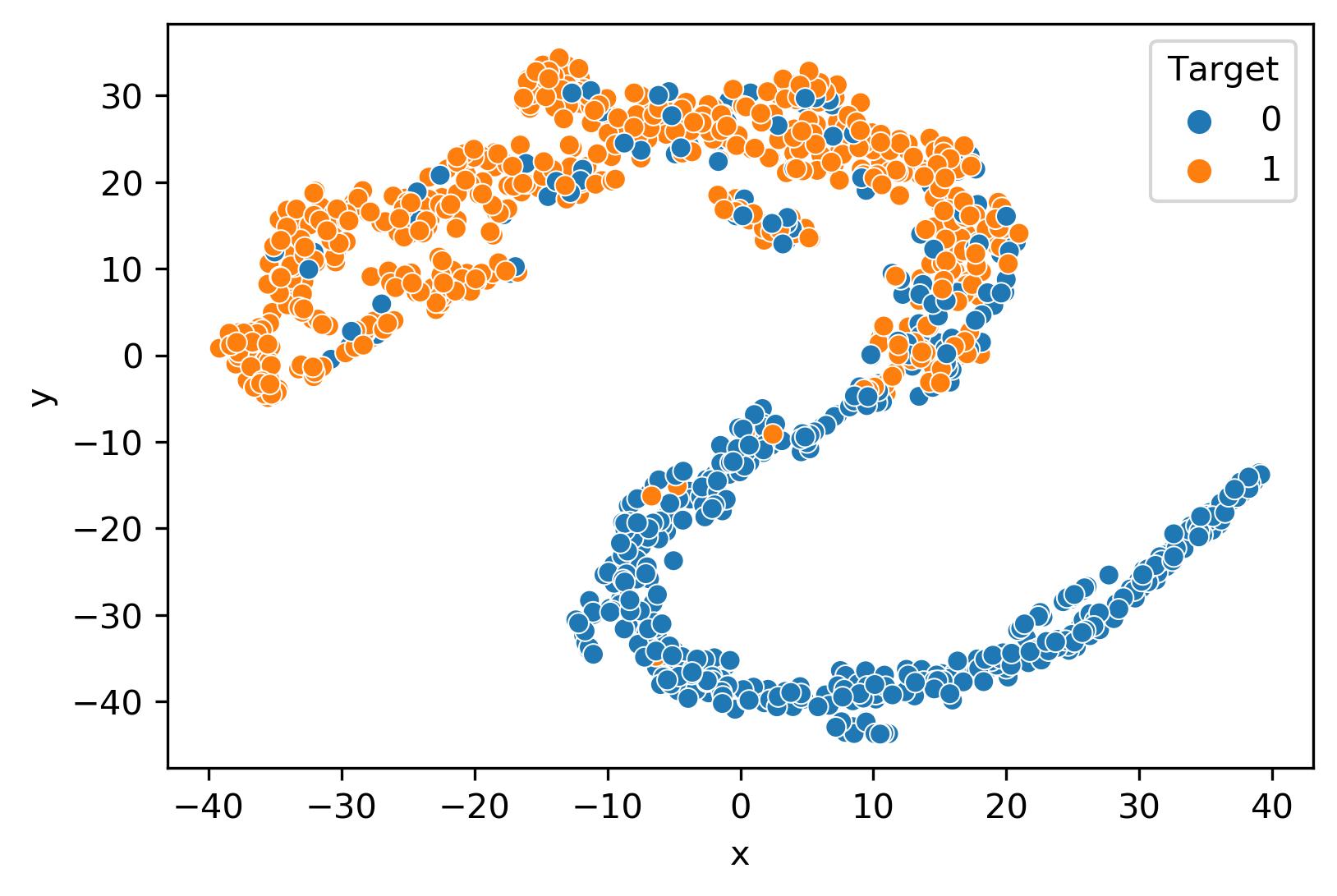

Supplement: btae643_Supplementary_Data [file btae643_supplementary_data.zip › Supplementary_Figure_3C_1200_dpi.jpg]

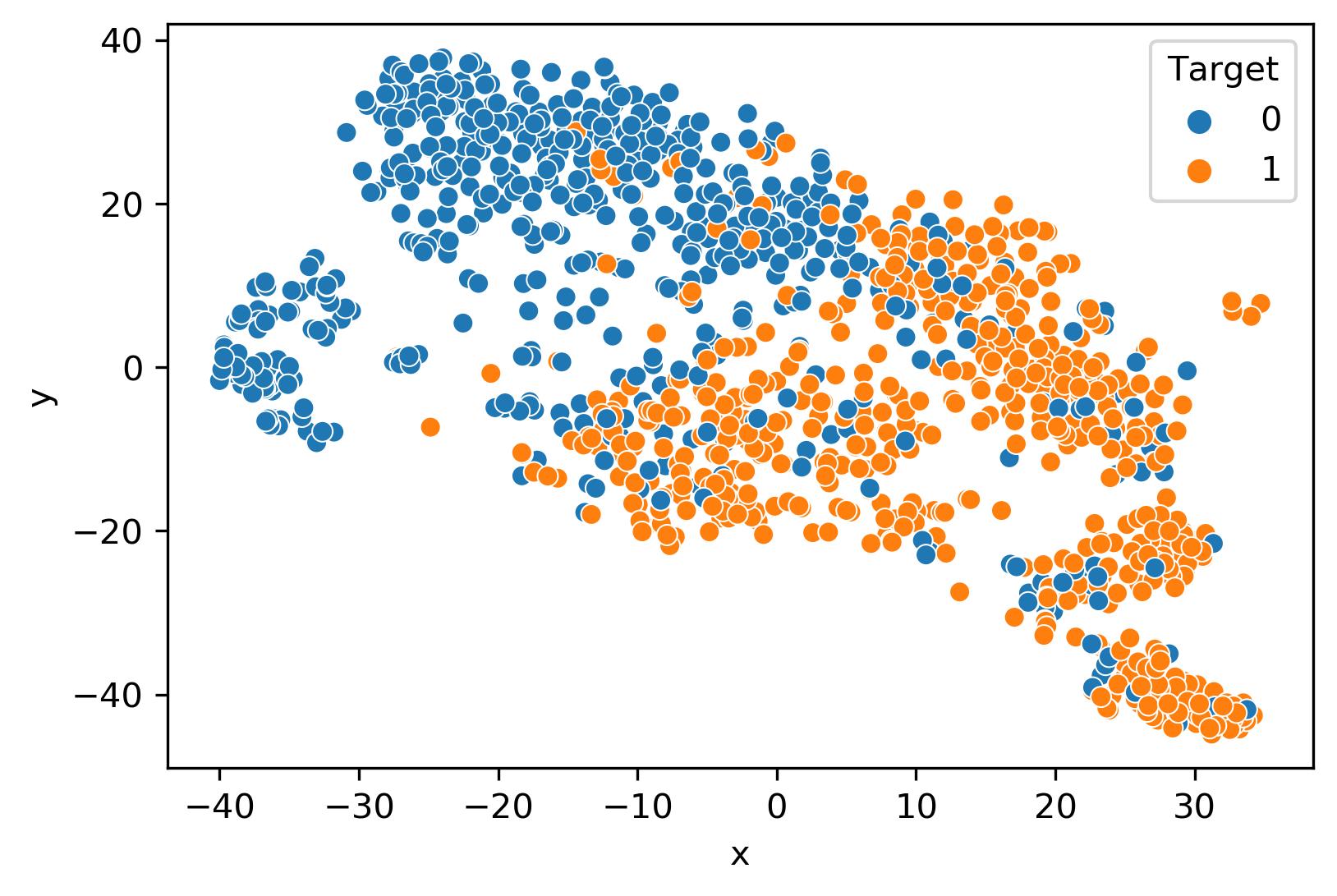

Supplement: btae643_Supplementary_Data [file btae643_supplementary_data.zip › Supplementary_Figure_3B_1200_dpi.jpg]
